# Community-based Mental Health Services in Norway

**DOI:** 10.17816/CP43

**Published:** 2021-03-20

**Authors:** Torleif Ruud, Svein Friis

**Affiliations:** Division of Mental Health Services, Akershus University Hospital; Clinic of Health Services Research and Psychiatry, Institute of Clinical Medicine, University of Oslo; Division of Mental Health and Addiction, Institute of Clinical Medicine, University of Oslo; Division of Mental Health and Addiction, Department of Research and Innovation, Oslo University Hospital

**Keywords:** community-based mental healthcare, mental health services, community mental health centres, community psychiatry, Norway, внебольничная психиатрическая служба, психиатрическая служба, местные центры психического здоровья, внебольничная психиатрия, Норвегия

## Abstract

Community-based mental healthcare in Norway consists of local community mental health centres (CMHCs) collaborating with general practitioners and primary mental healthcare in the municipalities, and with psychiatrists and psychologists working in private practices. The CMHCs were developed from the 1980s to give a broad range of comprehensive mental health services in local catchment areas. The CMHCs have outpatient clinics, mobile teams, and inpatient wards. They serve the larger group of patients needing specialized mental healthcare, and they also collaborate with the hospital-based mental health services. Both CMHCs and hospitals are operated by 19 health trusts with public funding.

Increasing resources in community-based mental healthcare was a major aim in a national plan for mental health between 1999 and 2008. The number of beds has decreased in CMHCs the last decade, while there has been an increase in mobile teams including crisis resolution teams (CRTs), early intervention teams for psychosis and assertive community treatment teams (ACT teams). Team-based care for mental health problems is also part of primary care, including care for patients with severe mental illnesses. Involuntary inpatient admissions mainly take place at hospitals, but CMHCs may continue such admissions and give community treatment orders for involuntary treatment in the community.

The increasing specialization of mental health services are considered to have improved services. However, this may also have resulted in more fragmented services and less continuity of care from service providers whom the patients know and trust. This can be a particular problem for patients with severe mental illnesses. As the outcomes of routine mental health services are usually not measured, the effects of community-based mental care for the patients and their families, are mostly unknown.

## THE MENTAL HEALTH SYSTEM IN NORWAY

Norway has a population of 5.4 million and 11 counties. Most people live in cities or towns, while the remainder live in small municipalities in scarcely populated, rural areas, with many having to travel long distances to access mental health services. The mental health services are public and are organized together with general health services at two organizational levels, with separate financing. The municipalities run the primary healthcare, including general practitioners (GPs), team-based primary mental health and substance abuse care. Hospitals and specialized mental health services were previously run by the counties. However, since a reform in 2002, they have been run by 19 health trusts, which are owned and instructed by four regional health authorities on behalf of the state, as owner of the specialized public health services.

The specialized mental health services for adults comprise CMHCs as secondary care; the clinics of mental health and substance abuse services with hospital wards and other specialized units, constitute tertiary care. Each health trust runs one or more general hospitals, including mental health clinics, and two or more CMHCs. The hospitals and CMHCs serve catchment areas with a defined population, but the service user is free to seek non-emergency healthcare outside the catchment area. Specialized psychiatric inpatient wards are in general hospitals and in buildings of former psychiatric hospitals. These consist of acute wards, specialized wards for psychosis, high security wards and departments for the elderly. Studies comparing CMHCs and hospitals have shown that patients with severe psychosis, severe aggressive behaviour or the need for coercion, were treated in hospitals, while patients with severe depression were divided equally between CMHCs and hospitals [1,2]. There are no forensic hospitals in Norway. Certain private service providers of mental healthcare are primarily financed through commissioning with the regional health authorities.

Reports from the Norwegian Patient Register have documented changes in the utilization of mental health services during the development of community-based, mental healthcare, and the data reported below have been sourced from three of these reports [2-4].

Parallel to the development of community-based mental healthcare, the use of specialized, inpatient mental health services per 100,000 adult inhabitants over the age of 18, has gradually been reduced by 53%, from 176 beds in 1998 to 86 in 2017. Of these, the number of hospital beds per 100,000 adult inhabitants totalled 91 in 1998 and 46 in 2017, while the number of CMHC beds was 81 (including 41 beds in psychiatric nursing homes) in 1998 and 36 in 2017 (when there were no remaining psychiatric nursing homes). During the last decade, 40% of the inpatient beds which provide mental health services for adults, have been in CMHCs.

The pattern of inpatient stays has also changed over the last 20 years, with an increase in the number of admissions (including an unknown increase of readmissions) and a decrease in the length of stays. The number of admissions per 100,000 adults was 860 in 1998 (620 in hospitals, 170 in CMHCs and 70 in psychiatric nursing homes) and 1290 in 2017 (690 in hospitals, 590 in CMHCs and 10 in other institutions). The combination of an increasing number of admissions, a reduction in the length of stays and a rather stable, total number of inpatients, indicates that the number of admissions per patient has increased. Local data in certain health trusts have identified a new group of “heavy users” with a high number of brief stays, which indicates that the increase in admissions per patient, varies among subgroups of patients.

Community-based mental healthcare must follow the same professional, ethical and judicial rules and regulations as other health services. National guidelines specify assessment and treatment for major patient groups, and special guidelines define priorities and acceptable waiting times for various mental disorders. New guidelines have now been developed for care pathways for major patient groups.

Child and adolescent mental health services are also part of the specialized health services, operated by the health trusts. Most patients are seen by the child and adolescent outpatient clinics, which are community-based clinics, usually with the same catchment area as a CMHC. Certain adolescents with severe disorders, may have treatment in adolescent inpatient units, however, children tend to be treated in their homes and rarely in inpatient units. Mobile teams meeting the child/adolescent and the family in their home, are part of the community-based child and adolescent mental health services.

Addiction and substance abuse services consist of outpatient clinics as a part of the mental health services in health trusts, and of primary health and social services run by the municipalities. The addiction and substance abuse departments in the health trusts also have inpatient wards, with various programmes for detoxification and treatment. With the high comorbidity of mental disorders and substance abuse problems, there has been an increasing emphasis on treatment of both types of problems concurrently by the same services. There are also several private addiction and substance abuse clinics, run by private organizations. The use of addiction and substance abuse services are not described further in this paper.

The total budget for the specialized mental health services for all age groups, was 48,000 euros per 100,000 inhabitants in 2017 [4]. This equated to 6.9% of the total health budget, which was 10.4% of the gross national product [5]. In Norway, the specialized health services are financed through the health trusts; GPs and primary healthcare are financed through the municipalities. This division of financing may increase the risk of fragmentation of services, as well as a lack of coordination if priorities are different at the two service levels.

GPs are an important part of primary healthcare. A GP reform in 2001 assigned each citizen to a GP of their choice, to secure accessibility and continuity of healthcare. The municipalities are also responsible for all primary healthcare, including primary mental healthcare and local substance abuse care. A reform in 2006 integrated social services, employment services and social security as services given by local units of the new governmental agency, New Employment and Welfare Administration (NAV). As the municipalities have GPs on call 24/7, and the primary care services are given in the home of the patient when necessary, primary care is an important part of the outreach services in Norway, even for patients with severe mental illness.

## THE DEVELOPMENT OF COMMUNITY-BASED MENTAL HEALTHCARE

In Norway, community-based mental healthcare is mainly provided by the CMHCs (referred to in Norway as the District Psychiatric Centres (DPS)), operating as part of the specialized mental health services, run by 19 health trusts. The CMHCs collaborate with the GPs and the primary mental healthcare services in the municipalities [6-8].

The development of the CMHCs in Norway started in the 1980s, by combining psychiatric outpatient clinics with local psychiatric wards (either new wards or nursing homes adapted to become active, long-term inpatient wards). The outpatient clinics were previously in general hospitals, along with intermediate-term inpatient wards for patients with more moderately severe mental disorders, following a hospital reform in 1950 [6]. Psychiatric nursing homes had also been established from the 1950s to provide care for patients with severe mental disorders, who still required care after treatment in psychiatric hospitals. The development of CMHCs was an important part of Norway's 10 years national Mental Health Plan 1999-2008, focusing on a major increase in funding and a revision of the organization of services. Other central features included increasing the specialized child and adolescent mental health services, and establishing primary mental healthcare teams, day centres and supported housing in the municipalities [9,10]. National guidelines for CMHCs were published in 2001 and 2006 [11,12].

The CMHCs established small, local inpatient wards for short term treatment. These were closer to home than the acute psychiatric and intermediate-term wards in the hospitals. In the 1980s, CMHCs began to develop generic, mobile, psychiatric rehabilitation teams, without a specified model. This development has continued over the last two decades, with more specific types of mobile teams serving special patient groups or purposes.

## COMMUNITY MENTAL HEALTH CENTRES

A CMHC serves the population in a local specific catchment area. The catchment areas of the current 66 CMHCs have an average population of 65,000 adults (range 20,000-130,000) adapted to geography and population density [3]. Each centre consists of outpatient clinics, mobile teams and inpatient wards. Inpatient CMHC wards, which are not common in other countries with CMHCs, offer short-term stays during crises, and longer stays for treatment and rehabilitation [8]. The number of CMHCs has increased from a limited number in the 1980s to 66 CMHCs throughout Norway, but with considerable variations in staffing and available services [13].

Altogether 3050 persons per 100,000 adult inhabitants, were CMHC patients in 2017, and of these 31% had an affective disorder, 36% had an anxiety (or related) disorder, 7% had a personality disorder and 9% had schizophrenia [3].

Outpatient clinics were developed from the 1950s when public mental health services were introduced in general hospitals. The purpose of these clinics was to serve patients with non-psychotic disorders, who were not served by the psychiatric hospitals, established over the last 100 years. The outpatient clinics increased in capacity and type of treatment in the following decades, and especially during the period of the national Mental Health Plan 1999-2008 and the subsequent decade. The number of annual outpatient consultations in mental health services increased threefold from 13,980 per 100,000 adults in 1998, to 41,860 in 2017 [2]. The percentage of outpatient consultations which were provided at CMHCs, increased from 53% to 86%, showing that only a small proportion of outpatients are currently being treated in hospitals. Psychiatrists and clinical psychologists in private practice also provide outpatient treatment, and they have financial support from the regional health authority. In 2018, there were six psychiatrists and 12 clinical psychologists working in private practices per 100,000 inhabitants [14]. In 2017, the number of outpatient consultations with psychiatrists and psychologists in private practices was 15,060 per 100,000 adults.

The growth in CMHC resources during the last decade, has mainly occurred in mobile teams, and in 2017 the number of team members per 100,000 adult inhabitants was 20 in the mobile teams, compared to 65 in outpatient clinics. Early intervention teams for psychosis were established in many CMHCs, inspired by a Norwegian research project [15,16], and in many CMHCs these teams were organized together with teams for the long-term treatment of individuals with psychosis. CRTs have been established in almost all CMHCs in Norway [17,18], and they meet twice a year in a national network for acute mental health services. A multi-centre study reported the implementation of the CRT model, but also demonstrated a considerable variation in team practices. However, overall, a significant clinical improvement was noted during crisis intervention, and with a high level of patient satisfaction [19]. An earlier study indicated that CRTs with extended opening hours, may prevent admissions for patients with moderately severe mental disorders [20]. The first 12 ACT teams established in Norway, were implemented well [21] and contributed to a great reduction in the use of inpatient wards [22] and to highly satisfied patients [23]. During the last few years, many new Functional ACT teams (FACT teams) have been established, based on a model from the Netherlands [24]. This model integrates individual and less intensive case management during stable periods, and a more intensive ACT team follow up during periods of crisis. This may provide better continuity of care from the same team across stable and unstable periods; and with a wider target group, the FACT teams may serve a larger number of patients and be more suitable in areas with lower population density.

The CMHCs have multidisciplinary clinical staff with psychiatrists, clinical psychologists, mental health nurses and several other professional groups. Psychiatrists/physicians and psychologists constitute 56% of the personnel in outpatient clinics and 29% in mobile teams, while mental health nurses and other staff make up 31% of the personnel in outpatient clinics, 64% in mobile teams and majority of the personnel in CMHC inpatient wards. In Norway, psychiatrists and clinical psychologists will have specialized in their specific discipline over a five-year period, after their six years of study in medicine or psychology. Mental health nurses and other professionals will have spent four years in education at university colleges, but they are also increasingly expected to have a master’s degree.

Several institutes and training programmes have offered further training for clinicians and health workers in community-based mental health services. These include institutes and training programmes in various types of psychotherapy and group therapy, as well as in treatment of specific patient groups. One large national programme with governmental support has, over the last 20 years, run two-year, local, multidisciplinary clinical training programmes throughout Norway, for health workers in CMHCs and primary care, working with patients with severe mental illnesses in community-based mental healthcare [25,26].

CMHCs are expected to assess and treat patients with the most common mental disorders, including severe mental illnesses. Patients needing more specialized outpatient treatment or inpatient treatment in wards with special competence or higher staffing, may need a referral to other departments in the health trust or for treatment at the regional level. One challenge is to encourage the use of mental health services among immigrant populations. In spite of a slightly higher prevalence of mental problems, immigrants use such services less than native Norwegians [27,28].

The type of assessments and treatments given by the CMHCs have become more differentiated and comprehensive, with growing staffing and with an increasing number of models for specific assessments and treatments for different groups of patients. In larger CMHCs with higher staffing and greater population numbers in the catchment area, there may be specialized teams or programmes for assessment and treatment of early psychoses, bipolar disorders, personality disorders, obsessive compulsive disorders or eating disorders. A network of CMHC teams for day treatment and group treatment of individuals with personality disorders, has documented the treatment effect of such teams in many CMHCs [29]. This is an example of a network between similar teams across CMHCs.

The CMHCs are secondary care services, interacting and collaborating both with GPs, the primary care teams of the municipalities and with tertiary hospital services. Clinicians at many CMHCs visit GPs and the primary care teams, either on a regular or an ad hoc basis for collaboration or to have joint meetings with patients. Many of these meetings are organized in so-called “responsibility groups” where individuals from various services meet a patient to plan and coordinate services for him or her. Demands from health authorities to documented efficiency (such as the high number of sessions or short waiting times) are limiting the amount of time that clinicians in the CMHC may use visiting primary care. The CMHCs also have regular or ad hoc interaction and collaboration with various hospital wards, for the mutual referral of patients with changing needs.

The national health authorities have put an increasing emphasis on the involvement of service users and their families in the development of community-based mental health services. User councils are now established in the health trusts, and user and family organizations receive financial support from the government. Clinicians are learning to focus more on personal recovery and patient preferences. However, there is still a long way to go before recovery-oriented services and shared decision-making with the patient, is routine practice for all clinicians in all CMHCs [30]. Patient-controlled brief admissions (self-referrals) to CMHC inpatient wards is a model innovated in a Norwegian CMHC [31], and this model was implemented in 52 of the 66 CMHCs, in 2017 [3].

The quality of the CMHCs has been rated in national surveys, in which GPs rated the local CMHC regarding workforce, competence, discharge letters, help in emergency situations and guidance for GPs. In 2006, the average CMHC quality was rated as medium, but with a large variation among CMHCs [32]. A similar national survey of outpatients’ experiences with the CMHCs showed medium to high positive associations with GP’s ratings of the CMHCs, and that patients were generally more satisfied than GPs [33]. The most important aspect for the patients was interaction with the clinicians and being met with respect. The most important factors for GPs were ensuring that the CMHC operated at an adequate level of competence, had a low rejection level of referrals and a short waiting time for patients. The effect of CMHCs and other community-based mental healthcare cannot be reported, as clinical outcome is not measured routinely in the mental health services in Norway [34].

## STRENGTHS AND WEAKNESSES OF THE COMMUNITY-BASED MENTAL HEALTHCARE

The CMHCs and other elements of community-based mental healthcare have greatly improved access to mental healthcare, by bringing the services closer to where people live, increasing the capacity of the services and developing more differentiated and comprehensive services to meet a diversity of needs.

However, surveys and resource statistics indicate that there are substantial variations in capacity and the quality of care across CMHCs. Leaders and clinicians must use effective strategies to identify local needs for improvement and to apply necessary implementation support, to change clinical practice and to provide more beneficial and effective mental health services.

Increasing specializations contribute to more comprehensive assessments and treatments of mental disorders. However, increasing specialization may also contribute to more fragmented services and less continuity of care. Pressure to increase efficiency by shortening treatment time in mental health services, may be a threat to developing and maintaining a trusting relationship between a patient and a clinician. This is especially important for patients with severe mental illnesses, the recovery of whom may take several years, and where the continuity of a long-term relationship with one person is often a key factor. Several recent studies on patient experiences have shown the importance of such long-term relationships and continuity of processes [35-37].

Community-based mental healthcare in Norway is serving a large group of individuals with mental health disorders, but most of the resources are still spent on inpatient services. Compared to several other countries with highly developed community-based mental healthcare, Norway uses more resources in inpatient services [7]. Analysing our health system and learning from other countries may show how we could use our resources in an even better way.

## FUTURE DEVELOPMENTS

Current national health policies aim to increase the percentage of healthcare given by primary care, and to increase competence in primary care. An ongoing merging of small municipalities into larger ones, may increase resources and the possibility that primary care may be able to take on larger responsibilities. It has also been suggested to pilot a transfer of CMHCs to primary care, by allowing certain larger municipalities to run CMHCs. However, many people in the CMHCs are worried about this possible development, and fear that the quality of the services will be lower.

In rural areas, with a low population density and long travelling times to access mental health services, web-based portals and other types of electronic communications with patients, are already being used by certain CMHCs in assessment and treatment. These forms of communication will probably be increasingly used in the future [38].

## Authors contribution

Torleif Ruud: planning, researching literature, obtaining data and writing the article; Svein Friis: writing the article.

## Conflicts of interest

The authors declare no conflict of interest.

## Financing

The article was written without any external funding.
